# The burden of smoking in Israel–attributable mortality and costs (2014)

**DOI:** 10.1186/2045-4015-3-28

**Published:** 2014-08-29

**Authors:** Gary M Ginsberg, Haim Geva

**Affiliations:** 1 Medical Technology Assessment Sector, Ministry of Health, Jermiahu 39, Jerusalem 9446724, Israel; 2 Department of Health Promotion, Ministry of Health, Jerusalem, Israel

**Keywords:** Mortality, Smoking, Smoking, Attributable mortality, Costs

## Abstract

**Background:**

Tobacco use is the single most preventable cause of death, incurring huge resource costs in terms of treating morbidity and lost productivity. This paper estimates smoking attributable mortality (SAM) as health costs in 2014 in Israel.

**Methods:**

Longitudinal data on prevalence of smokers and ex-smokers were combined with diagnostic and gender specific data on Relative Risks (RR) to gender and disease specific population attributable risks (PAR). PAR was then applied to mortality and hospitalization data from 2011, adjusted by population growth to 2014 to calculate SAM and hospitalization days (SAHD) caused by active smoking. These were used as a base for calculating deaths, hospital days and costs attributable to passive smoking, smoking by pregnant women, residential fires and productivity losses based on international literature.

**Results:**

The lagged model estimated active SAM in Israel in 2014 to be 7,025 deaths. Cardio-vascular causes accounted for 45.0% of SAM, malignant neoplasms (39.2%) and respiratory diseases (15.5%). Lung cancer alone accounted for 24.1% of SAM. There were an estimated 793, 17 and 12 deaths from passive smoking, mothers-to-be smoking and residential fires. Total SAM is around 7,847 deaths (95% CI 7,698-7,997) in 2014.

We estimated 319,231 active SAHD days (95% CI 313,135-325,326). Respiratory care accounted for around one-half of active SAHD (50.5%). Cardio-Vascular causes for 33.5% and malignant neoplasms (13.2%). Lung cancer only for 4.6%. Total SAHD was around 356,601 days including 36,049 days from passive smoking. Estimated direct acute care costs of 356,601 days in a general hospital amount to around 849 (95% CI 832–865) million NIS ($244 million). Non acute care costs amount to an additional 830 million NIS ($238 million). The total health service costs amount to 1,678 million NIS (95% CI 1,646-1,710) or $482 million, 0.2% of GNP. Productivity losses account for a further 1,909 million NIS ($548 million), giving an overall smoking related cost of 3,587 million NIS (95% CI 3,519-3,656) or $1,030 million, 0.41% of GNP).

**Conclusions:**

Smoking causes a considerable burden in Israel, both in terms of the expected 7,847 lives lost and the financial costs of around 3.6 million NIS ($1,030 million or 0.42% of GNP).

## Background

Tobacco use is the single most preventable cause of death [1], associated with more than five million deaths annually worldwide [2]. By 2030, tobacco related mortality is likely to rise to more than 8 million people [2]. Up to half of the world’s more than one billion smokers will die prematurely of a tobacco-related disease [2]. Therefore, reducing the burden of disease from smoking is obviously of paramount importance in the field of public health.

In Israel, 2014 mid-year population 8,227,200 [3], smoking prevalence among Jews has declined over the period 1980-1997-2010 from 45.0%-32.4%-23.9% among males and from 30.9%-25.0%-16.0% among females [4]. Amongst the non-Jewish population the decline has been far less over the period 1997–2010 from 46.9% to 43.4% among males, with a slight rise from 5.5% to 6.5% among females.

In Israel, the first estimate of smoking attributable mortality (SAM) was made for the 2001 National Gillon Commission to decrease damage from smoking [5]. This estimated 9,527 deaths from active smoking in 1999 and a further 1,385 deaths from passive or enforced smoking by foetuses, children, spouses and workmates. These estimates assumed a nine year lag period for each diagnosis, ignored elevated risks in ex-smokers and did not use age-specific risk categories.

An improved SAM estimate of 8,664 deaths attributable to active smoking in 2003 was published [6], based on the expansion of categories in the US Centres for Disease Control [7] on-line user-friendly computational program, called SAMMEC (Smoking Attributable Mortality, Morbidity, and Economic Costs). This estimate was based on

a) SAMMECs diagnosis list [7] which in turn was based on the 2004 US Surgeon General’s Report [8].

b) The enormous body of US and international literature on health risks due to tobacco use, which includes major studies [9] and compendiums of studies [10]. This was in order to correct for omitted diagnoses in the SAMMEC list such as diabetes which had subsequently been proven to be smoking-related [11].

More importantly, the estimates corrected for the fact that the standard SAMMEC approach did not consider the latency period that occurs between exposure to the risk factor (tobacco) and tobacco-induced mortality. The estimates also expanded age categories to include persons under 35 years old (who were excluded from the SAMMEC estimates).

In 2007, SAM from active smoking was estimated to be 8,932 deaths [5] using similar methodology to the 2003 estimates [6].

At the request of the ministry’s public health department, we were asked to prepare estimates of mortality and monetary costs attributable to smoking in 2014 in Israel for inclusion into the minister’s report to parliament on smoking in Israel, which was published in May 2014 [4].

## Methods

Historical prevalence data on smokers was based on data bases of the Ministry of Health’s health promotion department, National Health Surveys carried out by the Central Bureau of Statistics (1996–7 and 1999), bi-annual national telephone surveys (starting in 1994, the latest one for 2013 being based on 6,014 respondents) and National Health Surveys carried out by the Israeli Center for Disease Control (2003–4 and 2007–2010) [4]. The basic methodology, including updated prevalence surveys for current and ex-smokers by age and gender [4], mortality rates and relative risk (RR) estimates is described in detail an earlier paper [6]. Smoking prevalence data for 2014 was estimated by applying the relative decrease in gender and religion specific prevalence rates from 2012 to 2013 to the period 2013 to 2014. These included not only persons who reported smoking cigarettes but also those who reported using other tobacco products (like the hookah).

All calculations were based on an estimated mid-year population in 2014 of 8,227,200 persons [3]. Cost were at 2014 price levels using an average exchange rate for the first half of 2014 of 3.481 NIS to the US dollar [14].

The surgeon general’s report of 2014 [13] was used to provide the widely acceptable rubric of disease categories providing up to date estimates of RR. Since it contained many highly aggregated categories (other cancers, other heart diseases, other vascular diseases), we dis-aggregated the categories using values in the SAMMEC categorization [7]. Meta-analyses of studies were used to provide RRs for peptic ulcers [14-16], Crohn’s disease [17-21] and ulcerative colitis in former smokers [17-19,21-23]. To complete the list of diagnoses (Table 1), we added the specific (protective effects) of Parkinson’s disease [24-26], endometrial cancer [27-29] and ulcerative colitis in current smokers [17-23].

**Table 1:** Relative risk (RR) of SAM by smoking status, diagnosis and gender

**Refs.**		**Males RR current**		**Females RR current**	
			**RR former**		**RR former**
	**Cancers:**				
[7]	Bladder	3.27	2.09	2.22	1.89
[7]	Cervical	na	Na	1.59	1.14
	Endometrial	na	Na	0.74	0.88
[7]	Esophageal	6.76	4.46	7.75	2.79
[7]	Larengeal	14.60	6.34	13.02	5.16
[7]	Leukemia	1.86	1.33	1.13	1.38
[13]	Lung	22.25	6.06	21.15	5.75
[7]	Lip, bucal, pharynx	10.89	3.40	5.08	2.29
[7]	Pancreatic	2.31	1.15	2.25	1.55
[7]	Renal	2.72	1.73	1.29	1.05
[7]	Stomach	1.96	1.47	1.36	1.32
[13,30]	Unspecified	2.31	1.41	1.83	1.26
[13,30]	Other cancers (a)	4.16	1.83	1.06	1.01
	**Respiratory:**				
[13]	Tuberculosis	3.49	1.92	2.51	2.32
[13]	Bronchitis	19.66	5.67	19.67	7.55
[13]	COPD inc emphysema	21.08	5.97	19.09	8.05
[13]	Pneumonia	3.99	1.96	2.21	1.68
[13,31]	Asthma adults	1.99	1.26	2.18	1.48
[13,30,32]	Other respiratory (b)	1.94	1.25	3.09	2.17
	**Vascular:**				
[13]	Rheumatic heart disease	2.17	1.17	2.01	1.18
[7]	Aortic aneurism	6.21	3.07	7.07	2.07
[13]	Coronary artery disease	2.87	1.55	3.03	1.49
[13]	Coronary heart disease	2.71	1.51	3.01	1.49
[13,30]	Cardiac dysrhythmias	4.29	1.93	4.29	1.66
[13,30]	Myocardial infarction	2.01	1.31	8.84	3.44
[13,30]	Peripheral vascular disease	7.73	3.03	7.73	3.09
[7,13,30]	Other heart diseases (c)	2.17	1.25	2.02	1.19
	Cerebrovascular disease				
[13]	Ages 35-64	2.47	1.35	2.15	1.35
[13]	Ages 65+	1.74	1.16	1.85	1.26
	**Digestive system:**				
[17-21]	Crohn’s disease	1.70	1.22	1.70	1.22
[14-16]	Peptic ulcers	2.00	1.28	2.36	1.39
[17-23]	Ulcerative colitis	0.70	1.55	0.70	1.55
	Endocrine-metabolic				
[13]	Diabetes	1.86	1.31	1.62	1.31
	**Other:**				
	Parkinson’s disease	0.37	0.76	0.36	0.69
	(a) includes breast, respiratory in situ, renal pelvis and vulvar				
	(b) includes cough, dsypnoea and other respiratory diseases				

### Calculation of SAM due to active smoking

We assumed persons who died younger had shorter lag-times (between the act of smoking and SAM) than persons who died at an older age [6]. Estimates of the lag-time where made by subtracting the disease and gender-specific average age at death by the gender-specific average age of smokers.

We assumed a lag time for all diagnoses of 2.5, 5, 7.5 and 10 years for persons aged 20–24, 25–29, 30–34 and for, 35–39-year-olds, reflecting the fact that the lengthy lag-times for active smoking (e.g. 25–40 years) are non-feasible in young persons.

A disease-specific age-related linear factor was applied to the 40–54-year age group (i.e. with lower than average time-lag). The average disease-specific lag-time was assumed to occur in the 55–59-year-age group. The average lag time estimates that we used varied from 23.7 years from brain cancer to 40.4 years for coronary artery disease.

A disease specific age-related linear factor was also applied to the over 60 age group (i.e. with increasing above average lag-time) within the constraints of achieving the overall average disease and gender-specific time lag. Next, for each diagnostic, age and gender group, smoking prevalence was obtained for the year relating to 2014 less the lag-time.

Data on RR was combined with lagged smoking prevalence data based on the following formula applied to each diagnosis, age and gender category and finally aggregated [7]:

SAM = Number who died in each category x Smoking Attributable Fraction (SAF)

where

$$\begin{array}{ll} \mathrm{SAF}\;= & \left[\left(p0\;+\;p1\left(\mathrm{RR}1\right)\;+\;p2\left(\mathrm{RR}2\right)\right)\;-\;1\right]/\\ & \left[p0\;+\;p1\left(\mathrm{RR}1\right)\;+\;p2\left(\mathrm{RR}2\right)\right] \end{array}$$

and

P0 = Percentage of never smokers

P1 = Percentage of current smokers

P2 = Percentage of former smokers

RR1 = Relative risk of death for current smokers relative to never smokers

RR2 = Relative risk of death for former smokers relative to never smokers

### Calculation of SAM due to passive smoking and residential fires

Deaths caused by passive smoking in Israel were estimated by adjusting the US figure [13] of 9.44% of active SAM by the relative differences in smoking prevalence between the USA (males 16.7%, females 13.6%) [13,33] and Israel (males 24.9%, females 12.6%) in 2010 [4,34].

In order to estimate foetal SAM in Israel, USA data showing foetal SAM to be 0.232% of active SAM [13] was adjusted

a) By the ratio of the Israeli National Smoking prevalence in pregnant women of 6.82%, (as a result of 46.5% of pregnant women stopping smoking) [34] to the USA figure of 8.43% [35].

b) By Israel’s 30.6% higher birth rate [36,37] to give an estimate that foetal SAM accounts for 0.244% of active SAM in Israel.

Deaths caused by residential fires related to cigarette causes was estimated by adjusting the US figure [13] of 0.14% of active SAM deaths by the relative differences by the smoking prevalence between the USA and Israel

### Cost of treating smoking-attributable morbidity

The initial morbidity cost estimates includes only of the direct costs of acute care viewed from a “narrow” health services perspective as there are no readily available data in Israel on costs that fall outside the health system, such as work absences, transportation to receive treatment and out-of-pocket expenses.

Active Smoking Attributable hospitalization days (SAHD) were calculated in a similar way to acute SAM estimates. These were based on actual numbers of diagnosis specific hospitalization utilization rates by age and gender from 2011, being applied to population data in 2014.

Hospitalization costs were calculated by summing:-

a) The product of the estimated number of hospitalization days in non-ICU units by the 2,251 New Israeli Shekels (NIS) per day tariff of the Ministry of Health [38]

and

b) Based on ICU accounting for 4.6% of adult non-obstetric general bed use [39], the product of ICU hospitalization days and 5,046 NIS, being 224% times the per-diem cost of non-ICU hospitalizations [39].

An approximation of the other non-acute direct costs (such as for medications, ambulatory care, nursing home care and rehabilitation) attributable to smoking was based on applying the latest estimate from the USA [13] showing that non-acute direct costs were 97.8% of smoking attributable acute care costs.

A further very rough approximation of “indirect costs” in Israel was based on USA data [13] showing that lost productivity costs alone, due to current and ex-smokers having higher rates of absenteeism than never smokers, to be 225% that of the costs of smoking attributable acute hospital care [13]. This impact was “dose related” in the sense that heavy smokers having higher levels of absenteeism than lighter smokers [40].

## Results

The updated lagged model estimated active SAM in Israel in 2014 to be 7,025 deaths (95% CI 6,875-7,175), after taking into account an estimated 174 and four fewer deaths, due to the protective effects of smoking on Parkinson’s disease and endometrial cancers respectively (Table 2). Cardio-vascular causes accounted for 45.0% of SAM, malignant neoplasms (39.2%) and respiratory diseases (15.5%). Six specific diagnoses alone accounted for over three-quarters of SAM:- Lung Cancer (24.1%), Coronary Artery Disease (18.5%), COPD including emphysema (13.0%), Myocardial Infarction (10.9%), Cerebrovascular Disease (6.1%) and Coronary Heart Disease (5.0%). Around 60.6% of SAM occurred in males.

**Table 2:** Active smoking attributable mortality-Israel 2014

	**Males**	**Females**	**Total**
**Cancers:**			
Bladder	155	27	182
Cervical	0	10	10
Endometrial	0	-4	-4
Esophageal	65	2	67
Larengeal	54	7	61
Leukemia	90	13	103
Lung	1127	562	1690
Lip, bucal, pharynx	29	11	39
Pancreatic	169	108	276
Renal	70	7	77
Stomach	109	23	132
Unspecified	43	23	67
Ureter	2	0	2
Other cancers	39	9	48
**Respiratory:**			
Tuberculosis	3	0	3
Bronchitis	6	4	9
COPD inc emphysema	522	394	916
Pneumonia	10	2	13
Asthma adults	10	22	32
Other respiratory	55	58	114
**Cardio-vascular:**			
Rheumatic heart disease	12	14	27
Aortic aneurism	60	30	90
Coronary artery disease	808	489	1297
Coronary heart disease	190	164	355
Cardiac dysrhythmias	36	23	59
Myocardial infarction	266	499	765
Peripheral vascular disease	54	77	132
Other heart disease	4	1	5
Cerebrovascular disease	230	200	430
**Digestive system:**			
Crohn’s disease	1	1	2
Peptic ulcers	2	3	5
Ulcerative colitis	-0.1	-0.1	-0.2
**Endocrine-metabolic:**			
Diabetes	115	80	194
**Other:**			
Parkinson’s disease	-80	-94	-174
Total	4256	2769	7025

Passive (or enforced smoking was estimated to account for an additional 11.29% of active SAM in Israel, amounting to estimated 793 further deaths. Exposure of the foetus to mothers smoking accounted for an estimated 0.24% of active SAM, or 17 additional foetal deaths. While residential fire accounted for an estimated 0.17% of active SAM, or 12 additional deaths. Therefore total SAM will be around 7,847 deaths (95% CI 7,698-7,997) in 2014 or 16.8% of overall expected mortality.

The model estimated that there were 319,231 (95% CI 313,135-325,326) active SAHD days in Israel in 2014 (Table 3). Respiratory care accounted for around one-half of active SAHD (50.5%). Cardio-Vascular causes accounted for 33.5% and malignant neoplasms (13.2%). Lung cancer accounted for only 4.6%. Around 63.0% of SAM occurred in males.

**Table 3:** Active smoking attributable hospital days-Israel 2014

	**Males**	**Females**	**Total**
**Cancers:**			
Bladder	7,419	883	8,303
Cervical	-	299	299
Endometrial	-	-308	-308
Esophageal	984	478	1,462
Laryngeal	1,519	298	1,817
Leukemia	2,154	300	2,454
Lung	9,392	5,156	14,548
Lip, bucal, pharynx	2,252	1,465	3,717
Pancreatic	1,530	1,059	2,589
Renal	2,206	166	2,372
Stomach	1,781	362	2,143
Unspecified	1,085	981	2,067
Other cancers	395	136	531
**Respiratory:**			
Tuberculosis	281	37	318
Bronchitis	4,499	7,520	12,019
COPD incl. emphysema	29,141	17,857	46,997
Pneumonia	44,697	16,441	61,137
Asthma adults	616	1,511	2,126
Other respiratory	20,618	17,904	38,523
**Cardio-vascular:**			
Rheumatic heart disease	522	664	1,186
Aortic aneurisms	3,071	553	3,624
Coronary artery disease	3,081	1,009	4,090
Coronary heart disease	3,064	1,084	4,148
Cardiac dysrhythmias	149	99	248
Myocardial infarction	12,306	13,369	25,676
Peripheral vascular disease	16,065	13,285	29,350
Other heart disease	3,975	908	4,883
Cerebrovascular disease	21,661	12,079	33,740
**Digestive system:**			
Crohn’s disease	700	384	1,084
Peptic ulcers	114	87	200
Ulcerative colitis	-16	-13	-29
**Endocrine-metabolic:**			
Diabetes	7,272	3,103	10,375
**Other:**			
Parkinson’s disease	-1,586	-931	-2,517
Total hospital days	200,948	118,225	319,173

There were an estimated 36,049 additional SAHD from passive smoking. A further 780 days were attributable to exposure of the foetus to mothers smoking and 541 due to residential fires caused by cigarettes. Therefore total SAHD will be around 356,601 (95% CI 357,908-371,915) days in 2014.

Estimated direct acute care costs of 356,601 days in a general hospital amount to around 849 (95% CI 832–865) million NIS ($244 million). An additional 830 million NIS ($238 million) is estimated for out-of hospital costs including ambulatory care, emergency room visits, out- patient visits and rehabilitation. The total health service costs are therefore 1,679 (95% CI 1,646-1,710) million NIS ($482 million) representing 2.6% of the health budget or 0.2% of GNP. Productivity losses account for a further 1,909 million NIS ($548 million, based on 225% that of acute care costs of 849 million NIS) giving an overall smoking related cost of 3,587 (95% CI 3,519-3,656) million NIS ($1,030 million) or 0.41% of GNP.

## Discussion

Smoking remains a huge preventable risk factor accounting for approx. 7,847 deaths in Israel (of which 7,025 are from active smoking alone). Approximately one death in every six is attributable to smoking. The monetary impact on health service resources and society amounts to around 3,587 million ($1,030 million), around 0.41% of GNP.

While Hypertension was originally included in the SAMMEC list [41,42], it has subsequently been dropped and also does not appear in the Surgeon-Generals list. However a recent longitudinal study of 5,512 Japanese males [43] found smoking to be an independent risk factor for hypertension with an adjusted RR of 1.13 (95% CI 1.03 – 1.23). Applying this risk factor to both genders would add an additional 55 deaths to our acute SAM (and additional 487 acute SAHD).

The decrease of active SAM from previous estimates is partly a result of the secular downward trend in smoking prevalence that has been continuing for the past 40 years, falling in males from 50.6% in 1974 to 36.2% in 1994 and 25.2% in 2013, and from 25.1% to 20.35 and 12.7% among females over the same period.

Another factor is decreases in the size of the RR of diseases such as diabetes, heart and cerebrovascular disease as reported by the US surgeon-general [13]. These however are partially offset by the reported increases in RR of lung cancers [13]. While accounting for nearly a quarter of all acute SAM, lung cancer only accounted for 4.4% of acute SAHD, since chemotherapy and radiotherapy are usually carried out on an out-patient basis, with cancer patients being hospitalized for surgery and hospice care.

The main limitation of the study is that we have made the implicit assumption that the RR which we used were based on data from studies carried out outside of Israel would also apply to the Israeli population. Of course there might be genetic or environmental factors that would result in different disease specific RR based on an Israeli population.

Secondly, the estimation of deaths and costs from passive, mothers smoking and residential fires is by necessity based on US data. Each one of these categories deserves separate calculations to be made based on Israeli specific data. However, due to time limitations, we think it is better to provide a guesstimate based on foreign data, than no estimate at all.

The reader should therefore regard the quality of estimates for active smoking as being good, but the estimates for from passive, mothers smoking and residential fires as being rough first-order estimates.

A recent national Israeli biomarker survey, indicated widespread exposure to environmental tobacco smoke (ETS) in the non-smoking Israeli adult population, especially among males, younger and less educated participants [44]. However the data on urine cotinine concentrations, based on a sample of only 248 persons, did not lend itself to enable estimations of mortality losses due to ETS in Israel. In addition, because good data on past ETS exposure are not currently available in Israel, we only were able to make a rough estimate of the harm due to second-hand smoke exposure based on adapting US data [13].

The resultant rough estimate of 787 deaths due to passive smoking could be an under-estimate or over-estimate as the potential relative exposure levels to passive smoking were just based on one year’s smoking prevalence data, instead of over a longer time period.

Also underestimated is the 12 deaths attributable to residential fires (0.175% of active SAM) caused by cigarettes since they do not include an estimate for non-residential fires.

The estimate of non-hospital related direct costs can be thought of as being conservative as it was based on being 97.8% of acute hospital costs [13]. This figure is lower than previously published estimates of 102.1% in Hong-Kong [45]. 102.5% in the USA [46], 107.5% in Germany [47], 113.2% in California [48] 135% in Taiwan [49], 161% in China in 2000 [50] and 273% in China in 2008 [51].

It should be noted that there are health impacts of smoking that do not have mortality consequences. The cost of these are implicitly included in our calculations, where we add 97.8% on to acute hospital costs for non-acute hospital care (such as for infertility on an outpatient basis).

For indirect costs, our estimate can again be thought of as conservative as it only contained estimates of smoking attributable lost productivity costs, thereby excluding transport costs, out-of-pocket expenditures and premature burial costs.

Smoking kills more Israelis than the combined mortality from obesity [52], lack of physical exercise [53], motor vehicle emissions [54], vehicle accidents, suicides and murders [36].

No one single intervention can totally reduce the considerable burden of disease from smoking. A multi-faceted approach is required, combining legislation, counter-advertising, taxation, prevention and cessation interventions [55].

Fortunately, many potential interventions to reduce the human and monetary burden from smoking have been identified and prioritized according to their cost-utility ratios in Israel. Many very cost-effective interventions have been identified in addition to interventions that are cost-saving (ie: where savings in treatment costs exceed the intervention costs). These include imposition of a higher tax on tobacco, Clonidine, Nortiptyline, varenicline (2 mg/day), Quitline Counseling and a combination of Medication and Quitline Counseling. Many interventions were found to be very cost-effective (having a cost per quality adjusted life year (QALY) less than GNP per capita):- Nicotine lozenges, Varenicline (1 mg/day), Nicotine patches with and without Nortipyline or Burropion, Buropropin, Nicotine Gum, Group counseling, Nicotine nasal spray, Individual Counseling, Nicotine inhaler [5]. Other potential interventions, not yet evaluated using cost-effectiveness analysis in Israel include novel cheap modes such as mobile phone text-messaging as well as pack warnings, 'plain’ packaging and smoke-free space regulations.

Unfortunately, Israel lags behind many developed nations in that it does not use cost-utility analyses to prioritize the adoption of new health technologies. There may be several reasons for this inadequacy. One reason may be insufficient appreciation within the Ministry of Health of the importance of cost-utility analysis and of recent advances in health economics which facilitate the integration of epidemiological and economic data. Another reason may be that Israel tends to be an early adopter of new technologies, with many new technologies being considered for inclusion in the benefits package well before all the data needed for cost utility analyses are available. However, early adoption of suitable technologies can be still further catalyzed if a-priori cost-utility analyses are carried out during the period between the conclusion of clinical trials and when manufacturers begin marketing the new technology.

Yet another reason for not using cost utility analyses may be a desire to ensure that the members of the benefits package committee (i.e. the committee that determines new entries into the basket of services) will have enough degrees of freedom to take into account considerations that are not captured in cost-utility analyses, such as uniquely Israeli values^a^. However, there is no contradiction between preserving some degrees of freedom for policymakers and providing them with sophisticated cost-utility analyses as key inputs into the decision-making process.

Accordingly, it behooves Israel to find ways to better incorporate cost-utility analyses into its prioritization processes, both with regarding to smoking interventions, and more generally. This could be done either by expanding the role of health economics within the Ministry of Health, or by establishing an institution akin to NICE, or by relying more heavily on non-governmental experts and research institutions.

We would also note that, in areas other than health care, Israel has established funded bodies like the “authority for the war against road accidents” and the “authority for the war against drugs”. The considerable mortality burden (around twenty times higher than that caused by traffic related mortality) attributable to smoking in this paper cries out for the establishment of a “national authority” to co-ordinate to identify and implement a multi-faceted intervention strategy to decrease the considerable burden from smoking in Israel.

## Conclusion

Smoking causes a considerable burden in Israel, both in terms of the expected 7,847 lives lost and the financial costs of around 3,587 million NIS ($1,030 million or 0.41% of GNP). Many cost-effective and cost-saving interventions exist that can reduce this huge burden on society.

## Endnote

^a^One such uniquely-Israeli value is the high priority given to fertility treatments. At the same time it should be kept in mind that in the case of most major illnesses – such as lung cancer, myocardial infarction, osteoporosis, stroke or diabetes – we are unaware of any uniquely Israeli values to be considered.

## Abbreviations

COPD: Chronic obstructive pulmonary disease; ETS: Environmental tobacco smoke; ICU: Intensive care units; GNP: Gross national product; MTA: Medical Technology Assessment; NIS: New Israeli Shekels; PAR: Population attributable risk; QALY: Quality adjusted life year; RR: Relative risk; SAHD: Smoking attributable hospitalization days; SAM: Smoking attributable mortality; SAMMEC: Smoking attributable mortality, morbidity, and economic costs; SIDS: Sudden infant death syndrome.

## Competing interests

The authors declare that they have no competing interests.

## Authors’ contributions

GG collected the data, built the model, co-ordinated the study and drafted the manuscript. HG collected data and contributed to the manuscript. Both authors read and approved the final manuscript.

## Authors’ information

Haim Geva Haspil has a masters degree in life science from Bar Ilan University. He works in the Health Promotion and Education Department of the Ministry of Health, as a senior coordinator of tobacco control. He is also a smoking cessation advisor in the I.D.F.

GARY GINSBERG holds an Msc (Econ) from London University and a DrPH from the University of North Carolina. He was previously employed by the WHO in Geneva. Currently, Dr. Ginsberg is Director of the Medical Technology Assessment Sector of the Israeli Ministry of Health. He specializes in evaluating potential interventions using Cost-Utility Analysis.

## References

[B1] The World Health Report 2002: Reducing risks, promoting healthy life 2002 Geneva: WHO 10.1080/1357628031000116808 14741909

[B2] WHO Tobacco Free Initative http://www.who.int/tobacco/health_priority/en/ Accessed 2nd February 2014

[B3] Central Bureau of Statistics, Monthly Abstract of Statistics, January 2014, CBS, Jerusalem, Israel http://www1.cbs.gov.il/publications14/yarhon0114/pdf/b1.pdf Accessed 10^th^ February 2014

[B4] Minister of Health’s Report on Smoking in Israel 2013. Public Health Services 2014 Ministry of Health (Hebrew). http://www.health.gov.il/PublicationsFiles/smoking_2013.pdf Accessed 9^th^ July 2014

[B5] Ginsberg G Rosen B Rosenberg E Cost-Utility Analysis Cost-Utility Analyses of Interventions to Reduce the Smoking-Related Burden of Disease in Israel. RR-540-10 2014 Jerusalem, Israel: Brookdale-Smokler Center for Health Policy Research

[B6] Ginsberg GM Rosenberg E Rosen L Issues in Estimating Smoking Attributable Mortality in Israel Eur J Public Health 2010 20 1 113 119 Epub 2009 Aug 19 10.1093/eurpub/ckp111 19692551

[B7] Smoking Attributable Mortality, Morbidity and Economic Costs (SAMMEC) http://apps.nccd.cdc.gov/sammec (Accessed August 15th 2014)

[B8] A report of the Surgeon General: Health Consequences of Smoking 2004 http://www.surgeongeneral.gov/library/smokingconsequences/ Accessed 10th October 2010

[B9] International Agency for Research on Cancer Monograph Volume 83. Tobacco Smoke and Involuntary Smoking 2004 Lyon, France: World Health Organization

[B10] Doll R Peto R Boreham J Sutherland I Mortality in relation to smoking: 50 years’ observations on male British doctors BMJ 2004 328 1519 1527 10.1136/bmj.38142.554479.AE 15213107 PMC437139

[B11] Foy CG Bell RA Farmer DF Goff DC Jr Wagenknecht LE Smoking and Incidence of Diabetes among US Adults, findings from the insulin resistance atherosclerosis study Diabetes Care 2005 28 2501 2507 10.2337/diacare.28.10.2501 16186287

[B12] Bank of Israel Bank of Israel http://www.boi.org.il/en/Markets/ExchangeRates. Accessed 8^th^ July 2014

[B13] US Surgeon General: The Health Consequences of Smoking—50 Years of Progress A Report of the Surgeon General.U.S 2014 11 Rockville, MD: Department of Health and Human Services, Public Health Service, Office of the Surgeon General 625 646

[B14] Kim JJ Kim N Lee BH Kang JM Seo P Lim MK Kwon JH Song BJ Lee JW Lee SH Park YS Hwang JH Kim JW Jeong SH Lee DH Jung HC Song IS Risk factors for development and recurrence of peptic ulcer disease Korean J Gastroenterol 2010 56 220 228 Korean 10.4166/kjg.2010.56.4.220 20962557

[B15] Rosenstock S Jørgensen T Bonnevie O Andersen L Risk factors for peptic ulcer disease: a population based prospective cohort study comprising 2416 Danish adults Gut 2003 52 186 193 10.1136/gut.52.2.186 12524398 PMC1774958

[B16] Kurata JH Nogawa AN Meta-analysis of risk factors for peptic ulcer: nonsteroidal antiinflammatory drugs, helicobacter pylori, and smoking J Clin Gastroenterol 1997 24 2 17 10.1097/00004836-199701000-00002 9013343

[B17] Mahid SS Minor KS Soto RE Hornung CA Galandiuk S Smoking and inflammatory bowel disease: a meta-analysis Mayo Clin Proc 2006 81 1462 1471 10.4065/81.11.1462 17120402

[B18] Carlens C Hergens MP Grunewald J Ekbom A Eklund A Höglund CO Askling J Smoking, use of moist snuff, and risk of chronic inflammatory diseases Am J Respir Crit Care Med 2010 181 1217 1222 10.1164/rccm.200909-1338OC 20203245

[B19] Castiglione F Diaferia M Morace F Labianca O Meucci C Cuomo A Panarese A Romano M Sorrentini I D’Onofrio C Caporaso N Rispo A Risk factors for inflammatory bowel diseases according to the “hygiene hypothesis”: a case–control, multi-centre, prospective study in Southern Italy J Crohns Colitis 2012 6 324 329 10.1016/j.crohns.2011.09.003 22405169

[B20] Lakatos PL Vegh Z Lovasz BD David G Pandur T Erdelyi Z Szita I Mester G Balogh M Szipocs I Molnar C Komaromi E Golovics PA Mandel M Horvath A Szathmari M Kiss LS Lakatos L Is current smoking still an important environmental factor in inflammatory bowel diseases? Results from a population-based incident cohort Inflamm Bowel Dis 2013 19 1010 1017 10.1097/MIB.0b013e3182802b3e 23399739

[B21] Higuchi LM Khalili H Chan AT Richter JM Bousvaros A Fuchs CS A prospective study of cigarette smoking and the risk of inflammatory bowel disease in women Am J Gastroenterol 2012 107 1399 1406 10.1038/ajg.2012.196 22777340 PMC3667663

[B22] Sicilia B Arribas F Nerín J López Miguel C Vicente R Gomollón F Risk factors for ulcerative colitis: A population-based, case–control study in Spain J Crohns Colitis 2008 2 158 161 10.1016/j.crohns.2008.01.003 21172206

[B23] Wang YF Ou-Yang Q Xia B Liu LN Gu F Zhou KF Mei Q Shi RH Ran ZH Wang XD Hu PJ Wu KC Liu XG Miao YL Han Y Wu XP He GB Zhong J Liu GJ Multicenter case–control study of the risk factors for ulcerative colitis in China World J Gastroenterol 2013 19 1827 1833 10.3748/wjg.v19.i11.1827 23555172 PMC3607760

[B24] Allam MF Campbell MJ Hofman A Del Castillo AS Fernandez-Crehuet NR Smoking and Parkinson’s disease: systematic review of prospective studies Mov Disord 2004 19 614 621 10.1002/mds.20029 15197698

[B25] Noyce AJ Bestwick JP Silveira-Moriyama L Hawkes CH Giovannoni G Lees AJ Schrag A Meta-analysis of early nonmotor features and risk factors for Parkinson disease Ann Neurol 2012 72 893 901 10.1002/ana.23687 23071076 PMC3556649

[B26] Kiohara C Kusuhara S Cigarette smoking and Parkinson’s disease: a meta-analysis Fukoka Igaku Zasshi 2011 102 254 265 21966751

[B27] Felix AS Yang HP Gierach GL Park Y Brinton LA Cigarette smoking and endometrial cancer risk: the role of effect modification and tumour homogeneity Cancer Causes Control 2014 25 479 489 10.1007/s10552-014-0350-1 24487725 PMC4151521

[B28] Al-Zoughool M Dossus L Kaaks R Clavel-Chapelon F Tinneland A Olsen A Overvad K Boutron-Ruault M-C Gauthier E Linseisen J Chang-Claude J Boeing H Schulz M Trichopoulou A Chryssa T Trichopoulos D Berrino F Palli D Mattiello A Tumino R Sacerdote C Bueno-de-Mesquita HB Boshuizen HC Peeters PHM Gram IT Braaten T Lund E Chirlaque M-D Ardanaz E Agudo A Risk of endometrial cancer in relationship to cigarette smoking: Results from the EPIC study Int J Cancer 2007 121 2741 2747 10.1002/ijc.22990 17657712

[B29] Zhou B Yamg L Sun Q Cong R Gu H Tang N Zhu H Wang B Cigarette smoking and the risk of endometrial cancer: a meta-analysis Am J Med 2008 121 501 508 10.1016/j.amjmed.2008.01.044 18501231

[B30] Howden LM Mayer JA United States Census Bureau. US Census Briefs. Age and Sex composition 2010 2011 http://www.census.gov/prod/cen2010/briefs/c2010br-03.pdf Accessed 4th February 2014

[B31] National Health and Nutrition survey 2009–2012 Ch 13. Smoking During Pregnancy. Pub Number 352, ICD,C 2014

[B32] Martin JA Hamilton BE Sutton PD Ventura SJ Menacker F Kirmeyer S Munson ML Division of Vital Statistics Births: Final Data for 2005. National Vital Statistics Reports 56 2007 6 18277471

[B33] Central Bureau of Statistics, Statistical Abstract of Israel 2014 – No. 65, CBS, Jerusalem, Israel 2014 http://www1.cbs.gov.il/reader/shnatonenew_site.htm Accessed 4th May 2014

[B34] Martin JA Hamilton BE Michelle JK Curtin SC Matthews TJ Division of Vital Statistics Births: Final Data for 2012. National Vital Statistics Reports http://www.cdc.gov/nchs/data/nvsr/nvsr62/nvsr62_09.pdf Accessed 15^th^ May 2014 25671704

[B35] Ministry of Health Price List http://www.health.gov.il/subjects/finance/taarifon/pages/pricelist.aspx. Accessed 15th May 2014

[B36] Ginsberg GM Kark J Einav S The price and value of resuscitation: Cost-Utility Analysis of Cardiac Resuscitation Services in Jerusalem 2014 Submitted to Resuscitation 10.1016/j.resuscitation.2014.10.024 25447040

[B37] Laaksonen M Piha K Martikainen P Rahkonen O Lahelma E Health-related behaviours and sickness absence from work Occup Environ Med 2009 66 840 847 10.1136/oem.2008.039248 19934118

[B38] Nelson DE Kirkendall RS Laweton RI Chrismon JH Merritt RK Arday DA Giovino GA Surveillance for smoking-attributable mortality and years of potential life lost, by state - United States, 1990 MMWR CDC Surveill Summ 1994 43 1 8 8208238

[B39] Shultz JM Novotny TE Rice DP Quantifying the disease impact of Cigarette smoking with SAMMEC II software Public Health Rep 1991 106 326 333 1905056 PMC1580242

[B40] Dochi M Sakata K Oishi M Tanaka K Kobayashi E Suwazono Y Smoking as an independent risk factor for hypertension: A 14-Year longitudinal study in male Japanese workers Tohoku J Exp Med 2009 217 37 43 10.1620/tjem.217.37 19155606

[B41] Levine H Berman T Goldsmith R Göen T Spungen J Novack L Amitai Y Shohat T Grotto I Exposure to tobacco smoke based on urinarycotinine levels among Israeli smoking and non-smoking adults: a cross-sectional analysis of the first Israeli human biomonitoring study BMC Public Health 2013 13 1241 10.1186/1471-2458-13-1241 24377966 PMC3879425

[B42] McGhee SM Ho LM Lapsley HM Chau J Cheung WL Ho SY Pow M Lam TH Hedley AJ Cost of tobacco-related diseases, including passive smoking, in Hong Kong Tob Control 2006 15 125 130 10.1136/tc.2005.013292 16565461 PMC2563564

[B43] Miller LS Zhang X Rice DP Max W State estimates of total medical expenditures attributable to cigarette smoking, 1993 Public Health Rep 1998 113 447 458 9769770 PMC1308416

[B44] Neubauer S Welte R Beiche A Koeneg HH Beusch K Leidl R Mortality, morbidity and costs attributable to smoking in Germany: update and a 10-year comparison Tob Control 2006 15 464 471 10.1136/tc.2006.016030 17130376 PMC2563685

[B45] Max W Rice DP Sung HY Zhang X Miller L The economic burden of smoking in California Tob Control 2004 13 264 267 10.1136/tc.2003.006023 15333882 PMC1747911

[B46] Yang MC Fann CY Wen CP Cheng TY Smoking attributable medical expenditures, years of potential life lost, and the cost of premature death in Taiwan Tob Control 2005 14 Suppl 1 62 70 10.1136/tc.2004.007963 PMC1766172 15923452

[B47] Sung HY Wang L Jin S Hu TW Jiang Y Economic burden of smoking in China, 2000 Tob Control 2006 15 Suppl 1 5 11 16723677 10.1136/tc.2005.015412 PMC2563547

[B48] Yang L Sung H-Y Zhengzhong M The-Wei H Rao K Economic costs attributable to smoking in China: update and an 8-year comparison, 2000–2008 Tob Control 2011 20 4 266 272 doi:10.1136/tc.2010.042028. Epub 2011 Feb 21 10.1136/tc.2010.042028 21339491 PMC3759015

[B49] Ginsberg G Rosen B Rosenberg E Cost-Utility Analysis Cost-Utility Analyses of Interventions to Prevent and Treat Obesity in Israel. RR-550-10. Brookdale-Smokler Center for Health Policy Research http://brookdale.jdc.org.il/?CategoryID=192&ArticleID=211 Accessed 10th May 2014

[B50] Ginsberg G Rosen B Rosenberg E Cost-Utility Analysis Cost-Utility Analyses of Interventions to Increase exercise in Israeli adults. RR-565-11, Brookdale-Smokler Center for Health Policy Research http://brookdale.jdc.org.il/Uploads/PublicationsFiles/RR-565-11-Physical-Exercise-English-report.pdf. Accessed 10th May 2014

[B51] Ginsberg GM Seeri A Fletcher E Koutik D Keresente E Shemer Y Mortality and morbidity from vehicular emissions in Tel-Aviv World Trans Policy Pract 1998 4 27 31

[B52] Farrelly MC Pechacek TF Thomas KY Nelson D The impact of tobacco control programs on adult smoking Am J Public Health 2008 98 304 309 10.2105/AJPH.2006.106377 18172148 PMC2376884

[B53] Fox K Merrill JC Chang H Califano JA Estimating the costs of substance abuse to the Medicaid hospital care programme AJPH 1995 85 48 54 10.2105/AJPH.85.1.48 PMC1615270 7832261

[B54] Phillips D Kawachi I Tilyard M The costs of smoking revisited NZ Med J 1992 105 240 242 1620500

[B55] Hocking B Grain H Gordon I Cost to industry of illness related to alcohol and smoking. A study of Telecom Australia employees Med J Aust 1994 161 407 412 7935093

