# Erratum to: The burden of smoking in Israel–attributable mortality and costs (2014)

**DOI:** 10.1186/s13584-016-0064-9

**Published:** 2016-02-10

**Authors:** Gary M. Ginsberg, Haim Geva

**Affiliations:** Medical Technology Assessment Sector, Ministry of Health, Jermiahu 39, Jerusalem, 9446724 Israel; Department of Health Promotion, Ministry of Health, Jerusalem, Israel

After publication of this study [1], the authors noticed an error in the Conclusions. It should be 3600 million NIS instead of 3.6 million NIS. Please see the correct Conclusions below:

## Conclusions

Smoking causes a considerable burden in Israel, both in terms of the expected 7847 lives lost and the financial costs of around 3600 million NIS ($1030 million or 0.42 % of GNP).
